# Lipid Peroxidation in Psychiatric Illness: Overview of Clinical Evidence

**DOI:** 10.1155/2014/828702

**Published:** 2014-04-27

**Authors:** Yash B. Joshi, Domenico Praticò

**Affiliations:** Department of Pharmacology, Center for Translational Medicine, Temple University School of Medicine, MERB 947, 3500 North Broad Street, Philadelphia, PA 19140, USA

## Abstract

The brain is known to be sensitive to oxidative stress and lipid peroxidation. While lipid peroxidation has been shown to contribute to many disease processes, its role in psychiatric illness has not been investigated until recently. In this paper, we provide an overview of lipid peroxidation in the central nervous system as well as clinical data supporting a link between lipid peroxidation and disorders such as schizophrenia, bipolar disorder, and major depressive disorder. These data support further investigation of lipid peroxidation in the effort to uncover therapeutic targets and biomarkers of psychiatric disease.

## 1. Introduction

Over the last century, few areas of clinical and biomedical inquiry have undergone as rapid a transformation as the neuroscience of psychiatric disorders. In the past several decades, increasingly sophisticated experimentation, investigational tools, and model systems have led to more nuanced approach to pharmacotherapy. Although different psychiatric disorders are currently thought to stem from unique abnormalities in neuronal biochemistry, circuitry, and/or brain architecture, emerging data indicates that oxidative stress is present and may play an active role in these psychiatric illnesses. In this paper, we will provide an appraisal of recent clinical findings on lipid peroxidation as it applies to the pathobiology of schizophrenia, bipolar disorder, major depressive disorder, and several other psychiatric disorders.

## 2. Lipid Peroxidation and the Central Nervous System

The degree of oxidative stress in the cellular milieu is a direct result of those processes that accelerate the production of reactive oxygen species and those that detoxify them (for a discussion on oxidative stress, see [1]). Reactive oxygen species such as superoxide anion and hydroxyl radicals are produced from a variety of cellular processes. These reactive oxygen species are neutralized by antioxidants such as vitamins C and E as well as enzymes such as superoxide dismutase (SOD), catalase, and glutathione peroxidase. Low levels of reactive oxygen species are utilized by redox sensors to modulate cell function, but high levels of reactive oxygen species damage and oxidize nucleic acids, carbohydrates, and lipids. The brain is approximately 2% of total body weight but utilizes 20% of total oxygen, allowing ample metabolic substrates for free radical generation [2]. In light of this, and given the fact that lipids are the major components of neuronal membranes as well as the myelin sheaths that help conduct neuronal signaling, their peroxidation and dysfunction can dramatically compromise brain function globally.

Thus far, brain lipid peroxidation is not able to be directly assessed in living subjects. In lieu of this, markers of global oxidative stress can approximate lipid peroxidation and include antioxidant status and the activity of antioxidant enzymes such as superoxide dismutase, catalase, and glutathione peroxidase peripherally [3]. More direct measures of lipid peroxidation* in vivo* include assessment of isoprostanes and malondialdehyde/thiobarbituric acid reactive species in a variety of biological fluids or alkanes such as pentane in exhaled air [3, 4]. Although circuit level dysfunction and the dynamic pathophysiology of psychiatric disorders are poorly approximated by postmortem investigation of the brain, such tissue analysis can aid in localization of lipid peroxidation in regions known to be important to psychiatric disease.

## 3. Schizophrenia

Schizophrenia is characterized by the presence of positive symptoms such as delusions, hallucinations, disorganized thinking, and negative symptoms such as flat affect. In part due to the fact that the success of treating the positive symptoms of schizophrenia with dopamine receptor antagonists and that intoxication with substances that increase brain levels of dopamine such as amphetamine or cocaine can lead to episodes of psychosis, inappropriate activation of dopaminergic pathways was initially thought to underlie the disease. However, this dopamine hypothesis of schizophrenia is unable to account for several clinical and pharmacologic observations, and other pathologic mechanisms are likely to play a role (for a lengthier discussion, see [5]). Among these mechanisms, the role of toxic metabolites resulting in oxidative damage has been proposed since the mid-1950s [6]. In their 1954 manuscript, Hoffer, Osmond, and Smythies purported that a highly reactive “adrenochrome” was involved in the evolution of schizophrenia symptoms. Although this adrenochrome hypothesis was quickly shown to be inadequate, oxidative insults to the brain in schizophrenia have continued to be explored.

The largest body of evidence of lipid peroxidation in schizophrenia is from indirect markers such as activity of SOD, catalase, and glutathione peroxidase. Increased peripheral activity of SOD has been observed in patients with schizophrenia, correlating with positive symptoms (hallucinations, delusions, disorganized behavior), and is reduced upon administration of antipsychotic medications [7, 8]. However, several reports have suggested that increased SOD activity occurs late in the disease, with reduced SOD activity early and in younger patients, implying SOD function varies with disease progression [9, 10]. Recently, study of SOD polymorphism in patients with schizophrenia has shown an association between the Ala-9Val variant and poorer performance in neuropsychological assessment [11]. Catalase activity in schizophrenia has been reported as increased, unchanged, or reduced by different investigators, although it is likely that peripheral levels poorly approximate brain levels of catalase especially since catalase activity can be dramatically altered by antipsychotic medications [12–14]. Finally, glutathione peroxidase has been generally reported to be reduced peripherally as well as in postmortem brains. Overall, these findings imply that there is a prooxidative state in schizophrenia. Assessment of more direct markers of lipid peroxidation has generally supported the more indirect assessments—patients with schizophrenia have greater plasma and cerebrospinal levels of thiobarbituric acid reactive substances, increased exhaled pentane, and increased urinary isoprostane-8-epi-prostaglandin F2 alpha [15–18].

Clinical interventions attempting to mitigate the effects of lipid peroxidation have been attempted including administration of eicosapentaenoic acid, long-chain omega-3 fatty acid, vitamin antioxidant (primarily vitamins E and C), and N-acetyl cysteine [19–23]. However, no placebo-controlled study to date has demonstrated long-term symptom improvement following normalization of lipid peroxidation. While some have reported that antipsychotic medications used for schizophrenia have altered markers of oxidative stress, others have found no improvement [24–26]. Interestingly, electroconvulsive therapy in schizophrenia patients reduces plasma thiobarbituric acid reactive substances, which is only evident months after the initial therapy [13].

## 4. Bipolar Disorder

Bipolar disorder is a mood disorder characterized by periods of both mania and depression. While its underlying pathophysiology remains multifaceted and elusive, recent data have indicated that mitochondrial dysfunction and aberration in oxidation status are important components of bipolar disorder. Andreazza and colleagues have previously shown that thiobarbituric acid reactive substances are elevated in both episodes of mania and depression and are even elevated compared to controls when in remission [27, 28]. Beyond a general prooxidative state, oxidative damage appears to also be localized in bipolar disorder. Using postmortem brain samples, Andreazza and colleagues have noted increased carbonylation and nitration along with isoprostane-8-epi-prostaglandin F2 alpha levels in the prefrontal cortex of bipolar disorder patients [29]. Additionally, oxidation also appears to localize to dopamine transporter-positive and tyrosine-hydroxylase neurons in the prefrontal cortex [30]. In depressed or euthymic females patients with bipolar disorder, sleep cycle disruption positively correlates with measures of malondialdehyde levels, a relationship not found in age-matched healthy controls which suggests that lipid peroxidation follows disease course rather than occurring incidentally [31]. Catalase and glutathione peroxidase levels are also elevated in patients with bipolar disorder during depressive episodes [32]. Treatment with the mood-stabilizer lithium reduces thiobarbituric acid reactive substances in those patients presenting for an initial manic episode as well as in those patients with episodes of hypomania (bipolar disorder type II) [32, 33]. Lithium administration in bipolar disorder patients has been noted to increase the activity of the Na+ K+ ATPase, a cellular event which is independently associated with reduction in lipid peroxidation [34]. Correlation between peripherally collected markers of lipid peroxidation and magnetic resonance imaging of bipolar disorder patients also explains the majority of variance in white matter lesions on diffusion tensor imaging in their brains. Longitudinal prospective trials that reduce oxidative burden in patients in addition to treatment with mood stabilizers would be instructive to see how much of the disease burden can be attributable to lipid peroxidation in bipolar disorder.

## 5. Major Depressive Disorder

Major depressive disorder is characterized by depressed mood and symptoms that significantly impair normal function. The mainstay pharmacologic therapy in major depressive disorder involves usage of serotonergic and noradrenergic agents for symptomatic control, but several observations have led to consideration of lipid peroxidation targets in the disease. For example, individuals who are depressed as young adults show increased rate of cardiac morbidity and mortality later in life and meta-analyses have indicated that patients who are depressed after myocardial infarction have worse cardiac outcomes that non-depressed patients [35, 36]. Since a major component of cardiovascular risk stems from lipid oxidation and atherosclerotic plaque progression, commentators have conjectured that elevated oxidative burden in major depressive disorder could account for this finding. In a recent study of community-dwelling elderly subjects, plasma levels of isoprostane-8-epi-prostaglandin F2 alpha were higher in those with depressive symptoms than in nondepressed controls [37]. These results have been observed in a younger cohort and other work has also indicated that increased urinary isoprostane-8-epi-prostaglandin F2 alpha is associated with depressed mood, especially in men [38, 39]. Increased glutathione peroxidase and superoxide dismutase levels are found in depressed patients, particularly those with chronic disease and these elevations persist for months even after initiation of standard pharmacotherapy [40, 41]. Thus, lipid peroxidation appears to be significantly perturbed in major depression, and its attenuation may be beneficial in reducing coincidental medical disease. As with the data in schizophrenia and bipolar disorders, longer-term studies must be carried out to better understand the role of lipid peroxidation in major depression.

## 6. Other Psychiatric Disorders

Evidence of lipid peroxidation has also been recently discovered in other psychiatric disorders. In a small study of adults with attention deficit hyperactivity disorder, serum malondialdehyde levels were elevated compared to nondiseased controls; however no correlation was found between symptom severity on neuropsychiatric battery and levels of malondialdehyde [42]. In children with attention deficit hyperactivity disorder, lipid peroxidation products are also found to be increased in urine when compared to controls [43]. By contrast, serum levels of malondialdehyde were found to correlate with behavioral assessment of severity of obsessive compulsive disorder [44]. Several reports have also described an association between other anxiety disorders and oxidative stress, including generalized anxiety disorder, panic disorder, and posttraumatic stress disorder [45–47]. Elevated markers of oxidation have also been found in brain regions of patients with autism spectrum disorder, including in temporal cortex and cerebellum [48–50]. Investigation of oxidation stress and lipid peroxidation in other psychiatric illness such as personality disorders and eating disorders, however, has not been extensively conducted.

## 7. Conclusion: Therapeutic and Diagnostic Implications

In summary, recent clinical data has revealed oxidative damage and lipid peroxidation is seen in several psychiatric disorders. Larger longitudinal studies must be conducted to see if measures of lipid peroxidation and oxidative stress can be used to determine risk of developing various psychiatric diseases and to see if long-term disease outcomes can be modified by interventions that mitigate reactive oxygen species. Assessment of oxidative damage and lipid peroxidation in patients is currently limited to using indirect peripheral assessment of brain lipid peroxidation or postmortem analysis of brain samples. Since lipid peroxidation appears to be present in several psychiatric disorders, measures of lipid peroxidation are unable to be used as specific biomarkers for screening or monitoring disease progression. Development of more sophisticated methods of detecting lipid peroxidation would undoubtedly be useful in this endeavor. Regardless, based on current evidence, further investigation of lipid peroxidation in psychiatric illness is likely to reveal clinically-relevant information and should be carried out.
